# Empowering people to be healthier: public health nutrition through the Ottawa Charter

**DOI:** 10.1017/S002966511400161X

**Published:** 2015-01-20

**Authors:** Mary A. T. Flynn

**Affiliations:** 1 Public Health Nutrition, Food Safety Authority of Ireland, Abbey Court, Abbey Street, Dublin 1, Republic of Ireland; 2 Faculty of Life and Health Sciences, University of Ulster, Cromore Road, Coleraine, BT52 1SA, Northern Ireland

**Keywords:** Ottawa Charter, Diabetes prevention, Childhood obesity, Calorie menu labelling, Evaluation

## Abstract

The WHO's Ottawa Charter highlights five priority areas for taking action in public health. Only one of them is at the individual level as action at more upstream intervention levels, such as community or policy levels, is critical for enabling individuals to succeed. The objective of the present paper is to give insight into the many complex processes involved in public health nutrition by describing the Ottawa Charter's five priority areas for taking action using public health nutrition initiatives I have been involved in. Evidence-based guidelines for healthy eating and infant feeding provide an essential basis for individuals to ‘develop personal skills’ (Action Area 1). ‘Re-orienting health services’ (Action Area 2) can address the needs of vulnerable population subgroups, such as the culturally sensitive diabetes prevention programme established for an Indo-Asian community in Canada. Identifying geographic areas at high risk of childhood obesity enables better strategic planning and targeting of resources to ‘strengthen community action’ (Action Area 3). Calorie menu labelling can ‘create supportive environments’ (Action Area 4) through encouraging a demand for less energy-dense, healthier food options. ‘Building healthy public policy’ (Action Area 5) to implement mandatory folic acid food fortification for prevention of birth defects has many advantages over a voluntary approach. In conclusion, evaluation and evidence-based decision-making needs to take account of different strategies used to take action in each of these priority areas. For this, the randomised control trial needs adaptation to determine the best practice in public health nutrition where interventions play out in real life with all its confounding factors.

Public health nutrition goes way beyond simply telling people what to do. While public health nutrition can be described as improving the health of populations through evidence-based nutrition, this belies the complicated processes involved. A list of descriptors have been compiled to communicate what public health nutrition is all about including: population-based nutrition; nutrition health promotion; use of food and nutrition systems; wellness maintenance through nutrition; nutrition for primary prevention; nutrition application using public health principles; nutrition education; environmental nutrition; politics and nutrition^(^^1^^)^. The present paper adds another definition and summarises public health nutrition as empowering people to be healthier through a wide range of strategies that enable healthy action and go much further than providing guidance on what actions to take. For example, consider the situations Mary and her family find themselves in, and the problems they face, as they try to overcome one of the commonest nutritional issues affecting children in our world today.

Mary is 7 years old. A few months ago on a routine visit to her Family Doctor, he took the unusual step of measuring her weight and height. Then he gently explained to Mary and her mother that her ‘weight growth was getting way ahead of her height growth’ – and that she needed to eat more healthily and be more active so that her height growth could catch up with her weight growth. While this was all very unexpected, the reassuring approach the Doctor took to explain how Mary could ‘grow into her weight’ encouraged and inspired both child and parent. Although only 7 years old, Mary was very relieved that someone had, at last, recognised she had a problem and was going to help her tackle it. Research shows children as young as age 4 years are not only aware of weight status, but have strongly negative attitudes to obesity^(^^2^^,^^3^^)^. Mary's mother's reaction was total surprise as she had not recognised that her child had a weight problem. This is very common among parents of overweight children because the constant changes in weight and height during childhood and adolescent growth, mask extra weight gain and make it very difficult to visually discern overweight^(^^4^^,^^5^^)^. Consequently weight status assessment of children and teenagers is a critical component of public health nutrition programmes, e.g. the National Child Measurement Programme^(^^6^^)^.

Despite being highly motivated Mary and her family are finding healthy eating and active living much harder than they anticipated. Consider a normal day in Mary's life to see what they are up against. As shown in Fig. 1 Mary spends about 5 h/d with her family, but that passes by in a ‘blur’ of homework/eat/ wash/dress/prepare meals/shop/tidy up etc. Most of Mary's day is spent in school and after-school-care but neither of these places has nutrition policies in place. Mary's healthy eating efforts set her apart from her classmates so she feels deprived and isolated during most eating occasions shared with children of her own age. To make matters worse, Mary's school, in common with many Irish primary schools, has a ‘no running’ policy in place for injury prevention^(^^7^^)^. This is where school rules prohibit running to prevent accidental injury and these rules extend to forbid running in the playground. Such rules prevent the physical activity that occurs spontaneously when primary school children are outside in a group. All of this makes Mary's efforts at healthy eating and active living very difficult, but these are not her biggest problems.

**Fig. 1.**
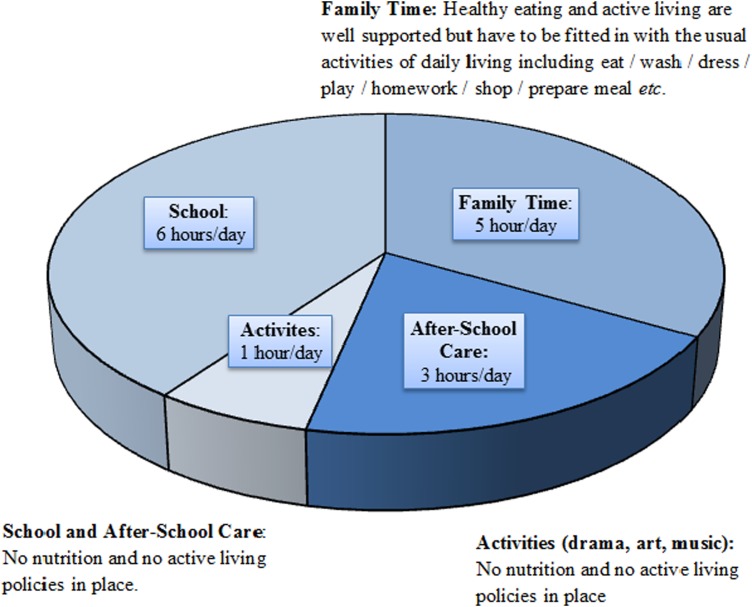
Healthy eating and active living opportunities during a typical day in the life of a girl aged 7 years (15 waking hours).

Mary finds trying to explain it all to her peers is the most challenging part. Friends are really important when you are age 7. What Mary is most worried about is what her best friend Jayne is going to make of it all next time they go to the movies. Jayne likes nothing better than a trip with Mary to the movies where they share the usual bucket of popcorn washed down with their very own jumbo-sized soft drink.

Every week Mary is discovering more challenges that make healthy eating and active living really big ordeals. She wishes for a world where things are switched around so that healthy eating and active living are easy and that, instead, people have to really struggle to have unhealthy lifestyle habits etc. then it would be simple, and probably even some fun too!

That is why public health nutrition has to go far beyond helping people to do their best and needs to intervene instead at more ‘upstream’ levels to enable people's best efforts to be successful. The purpose of the present paper is to give some insight into the complex processes involved in public health nutrition using the Ottawa Charter which outlines priority areas for action. A range of nutrition initiatives in Ireland and in Canada that I have been involved in are included, by way of examples, to describe the different strategies involved in practical terms.

## The Ottawa Charter

The Ottawa Charter was introduced at the first International Conference on Health Promotion, held in Ottawa in 1986 as a Charter for action to achieve health for all^(^^8^^)^. Introduced when the obesity epidemic was just starting, it is, perhaps, easier to see the relevance of the Ottawa Charter now, 28 years later, when all aspects of public health are affected by crisis levels of obesity. The Ottawa Charter outlines how action should be taken and it forms the basis of models that promote action in all areas of public health.

As shown in Table 1 the Ottawa Charter outlines five priority areas for action that are needed to improve population health. Of these only one is at the individual level (developing personal skills) as action at more upstream intervention levels such as re-orienting the health service, strengthening community action, creating supportive environments’ or building healthy public policy, is critical for enabling individuals to succeed.

**Table 1.** The Ottawa Charter for action to achieve health for all (WHO 21 November 1986)^(^^8^^)^

Build healthy public policy
Create supportive environments
Strengthen community action
Re-orient the health services
Develop personal skills

## Develop personal skills

Evidence-based guidelines for healthy eating and infant feeding provide an essential basis for individuals to ‘develop personal skills. This represents the only priority action area in the Ottawa Charter that focuses on telling people what to do. Guidelines formulated must reflect on available food, be aligned with cultural traditions, address problem areas and be acceptable. This requires research into the needs of target populations and special attention to those who are vulnerable or at risk of being excluded. Recent Work in developing the technical basis for policy on healthy eating and infant feeding guidelines for Ireland^(^^9^^–^^12^^)^ describes the evidence base required for this action area in public health nutrition. The particular importance of tailoring guidance so that actions advised are appropriate for, and truly enable, target populations is highlighted in the following two examples.

### Example 1: healthy eating guidelines

In an evaluation of healthy eating guidelines used in Ireland up to 2008, the need to provide practical guidance on overall energy intakes was identified i.e. advice on how much food is enough but not too much^(^^13^^)^. This involved developing specific guidance for different age and sex groups on food needs when following guidelines on healthy physical activity, i.e. being moderately active^(^^14^^,^^15^^)^ and when sedentary^(^^16^^)^. The carbohydrate food group was identified as a critical food group for providing guidance on energy needs for various activity levels. Consequently the guidance formulated on the number of servings required from this food group varied depending on the energy requirements of individuals. Advice was also given about how this needs day-to-day adjustment depending on activity levels. One of the biggest challenges encountered in formulating such guidance on energy intakes was the wide variation in portion size and energy content of the various foods included in the carbohydrate food group^(^^13^^)^. Some food servings from this food group just provided 293 kJ (70 kcal), whereas other provided more than 1046 kJ (250 kcal)^(^^13^^)^. Two approaches were explored to solve this.

1. All food portions were developed so that they provided the same energy (e.g. approximately 418 kJ (100 kcal)). This option provided portions that are simple to swap but had the major disadvantage of comprising only part of a natural food portion in most cases – e.g. one thin slice of pan sliced bread was equivalent to two-thirds of a thick slice or half a roll or one-third of a bagel or half a cup pasta or one-quarter cup of muesli etc.
2. A range of portion sizes were developed and categorised within four bands of energy content ranging from a minimum of 293 kJ (70 kcal) to a maximum of 921 kJ (220 kcal) (see Table 2). The main advantage of this second option was the maintenance of whole portion sizes for the majority of foods. However, a major disadvantage was the complication introduced where some foods only provided 418–565 kJ (100–135 kcal), whereas others provided 795–921 kJ (190–220 kcal).

**Table 2.** Guidance on adjusting energy intake using the carbohydrate food group: serving sizes for foods in the carbohydrate food group within four bands of energy content^(^^10^^)^

Calories	Cereals	Breads			Potatoes, Pasta, Rice		
Lowest 100–135 kcal	One-third cup raw porridge oats	One slice soda bread	One slice batch loaf	One medium bread roll	One medium boiled/baked potato	Three scoops mashed potato*	One cup cooked pasta**
Low to Mid 135–160 kcal	Two whole-wheat breakfast biscuits	Two regular slices pan bread	One oval pitta bread	Six wholemeal crackers	One cup sweet potatoes	One cup cooked basmati rice	
Mid to High 160–190 kcal	One-half cup muesli	One tortilla bread	One-half lunch size baguette	One-half panini bread	Eight baby potatoes	One cup cooked white rice	One cup cooked brown spaghetti
Highest 190–220 kcal	One and a half cups cereal flakes	Two thick-cut slices pan bread	One bagel	Two round pitta bread		One cup cooked brown rice	One and a half cup whole-wheat noodles

* Potatoes mashed with only low-fat milk added.

** One cup = 200ml disposable drinking cup.

Two food displays were developed outlining the two different approaches to portion sizes for carbohydrate foods and these were used to seek feedback from dieticians and nutritionists on which of the two food displays best represented average portion sizes. There was a unanimous agreement that the second option where a range of portion sizes were used (see Table 2) made most sense because people do not eat ‘bits and pieces’ of whole foods^(^^13^^,^^17^^)^. The same two food displays were used to seek feedback from over 1000 adult shoppers in advantaged and disadvantaged areas of Dublin. Over three quarters of shoppers preferred the second option of a range of portion sizes because these were closer to the portions they considered ‘average’ for the different types of food in this group^(^^13^^,^^17^^)^. Many consumers suggested that the option where all portions provided approximately 418 kJ (100 kcal) consisted of ‘parts of a portion’ that would possibly be appropriate for children but not for adults^(^^18^^)^. Consumers also pointed out that it was easy to limit portion sizes of foods that were ‘filling’, such as porridge or mashed potatoes, while this was much more difficult for foods that were not ‘filling’, such as rice or muesli. Some consumers (mainly men) described portions of these latter foods that were much bigger than the range of portions we developed up to the limit of 921 kJ (220 kcal)^(^^13^^,^^17^^)^.

This work demonstrates the importance of tailoring advice to suit the needs of the target population. In recent times misleading stories about the benefits of ‘low carb’ diets are common in the popular press and this adds to peoples’ confusion. The tendency to describe much bigger portion sizes (in excess of 921 kJ (220 kcal)) for carbohydrate foods that are not ‘filling’ reveals a basis for myths generated about how carbohydrate foods promote weight gain and obesity. The studies we conducted indicate that a range of portions sizes, along with information on which foods are ‘filling’ (i.e. porridge due to soluble fibre content) and which are not (i.e. white rice), is essential to help guide people how to eat enough but not too much food. As shown in Table 2 a good understanding of how these foods contribute differently to energy intakes is crucial for body weight control. The challenge for public health nutritionists is how to communicate such complex information in a way that is easily understood and simple to put into practice.

### Example 2: best practice for infant feeding

In addition to low breastfeeding rates, recent research on infant feeding practices in Ireland have highlighted suboptimal feeding practices concerning the types of solid food used to feed infants^(^^19^^)^. In the present study the authors outline how almost one third of 6-month-old Dubliners were reported to be consuming foods completely at variance with best practice infant feeding guidelines, such as ‘roast dinners with all the trimmings’, followed by chocolate pudding or cheesecake, accompanied by sugary drinks and biscuits^(^^19^^)^.

To address these poor weaning habits, public health nutritionists working on the formulation of best infant feeding practice guidelines interviewed mothers of young children (<30 months of age) about their own infant feeding practices and where they got their ideas for weaning foods^(^^20^^)^. Over two-thirds (68 %, *n* 113) of mothers surveyed used commercial baby foods, and 32 % (*n* 62) of mothers used the names of commercial infant foods as ideas for homemade meals. To investigate further, a scan of the nutritional composition of all the commercial infant foods marketed in Ireland was carried out. This identified 448 such foods available in Ireland. As many as 15 % (*n* 69) of these foods were not in line with best infant feeding practice, e.g. chocolate pudding, banoffi pie and strawberry cheesecake (all labelled as suitable from 4 months); biscuits, ‘roast dinners with all the trimmings’, sausage pasta bake (all labelled as suitable from 6 months) etc. (see Table 3). In all cases the nutritional composition provided on the label complied with European Union legislation governing baby foods because these rules do not extend to restrict high-fat, high-sugar and salty foods^(^^21^^)^. This shows that even for infants, parents and caregivers need to develop personal skills to enable them to choose suitable healthy foods from the array of foods commercially available, just as they do for older children, teenagers and adults.

**Table 3.** A scan of commercial baby foods marketed in Ireland (*n* 448): Examples of baby foods (with the age groups they are deemed suitable for) that are not in line with best infant feeding practice in Ireland^*^ (15 % *n* 69)^(^^20^^)^. (Re-produced with permission from The *Irish Medical Journal* 2012; 105(8): 267.)

Baby food (age deemed suitable for)	Reasons for inappropriateness
Chocolate Rice Pudding (from 4 months)	All of these foods are primarily based on added sugar and fat; In addition, food denoted ‘g’ contains gluten although marketed for infants younger than aged 6 months
Banoffi Pudding (from 4 months)	
Strawberry Cheesecake^g^ (from 4 months)	
Multigrain Biscuit (from 6 months)	
Apple Biscotti (from 6 months)	
Apple and Ginger Baby Cookies (from 6 months)	
Chocolate (from 9 months)	
Organic Gingerbread (from 9 months)	
Three Cheese Sauce (from 4 months)	These foods contain added salt
Beef Gravy (from 4 months)	
Diluted Orange and Apple Juice (from 4 months)	Breast milk or formula milk and water are the only recommended fluids for infants
Pure Apple Juice (from 4 months)	
Mixed Fruit with Mineral Water (from 4 months)	
Sausage Pasta Bake^g^ (from 4 months)	Contains a high salt processed meat; contains gluten although marketed for infants younger than aged 6 months
Lamb Roast Dinner with all the Trimmings (from 6 months)	Plain, bland food with minimal added fat or sugar and no added salt is recommended for infants

## Re-orient the health service

Work carried out in Canada demonstrates the impact of re-orienting the health service, the second priority area for action outlined in the Ottawa Charter. This work concerned the high risk of type 2 diabetes among the Indo-Asian community living in Calgary, Alberta (*n* 37 000).

Indo-Asians are particularly vulnerable to developing type 2 diabetes and this is evident in their countries of origin^(^^22^^)^ and in western countries to which they have emigrated^(^^23^^)^. This may be associated with several factors, including genetic predisposition, *in utero* metabolic factors and cultural practices (see^(^^24^^)^ for review). Type 2 diabetes also presents at a younger age in this population,^(^^25^^,^^26^^)^ but attendance at mainstream treatment and follow-up clinics is poor^(^^27^^)^. This combination is likely to contribute to the increased risk of developing both macrovascular and microvascular complications evident among Indo-Asians (see^(^^24^^)^ for review). Early therapeutic interventions for diabetes substantially reduce the risk of complications,^(^^28^^,^^29^^)^ whereas moderate lifestyle interventions in high-risk populations can prevent or delay the onset of type 2 diabetes^(^^30^^–^^32^^)^.

Despite increased risks associated with diabetes among the Indo-Asians, the majority of cases in this population group in Calgary at the turn of the millennium remained undetected and those that were identified were poorly managed due to non-attendance at mainstream clinics. It was felt that cultural barriers, including language difficulties, limited access to standard diabetes services for Indo-Asians and other Ethnic groups in Calgary^(^^27^^)^. This led the Calgary Health Region to re-orient the health service. In partnership with the leaders of the Indo-Asian community, a culturally sensitive, community-based diabetes risk screening programme was developed^(^^27^^)^. To overcome cultural and language barriers all risk screening procedures were performed by trained community volunteers at times and in places where the community gathered (mostly after weekly religious ceremonies in places of worship). Volunteers completed one-on-one interviews about diet and activity habits in English, Punjabi, Gujarati and Hindi, but recorded all data in English. Screening procedures also included anthropometric assessments and random capillary blood glucose tests. Over 900 adults with no history of diabetes attended the fourteen sessions held in various parts of the community. Blood sugar levels indicative of glucose intolerance were identified in over one-third (36 %) of those screened, two-thirds (67 %) were found to be overweight or obese and almost three-quarters (73 %) had excessive abdominal body fat^(^^27^^)^. Furthermore, dietary intakes tended to be high in energy and glycaemic carbohydrate foods, while only 27 % were sufficiently active^(^^27^^)^. More remarkable than the results of screening were the high numbers attending and the animated conversations they had with the volunteers who were supported by the public health nutrition team. Lively exchanges on how to improve the nutritional composition of traditional dishes through changes in food preparation, how to increase physical activity in culturally acceptable ways and how to manage diabetes and prevent diabetic complications all served to disseminate information that was reliable, appropriate and able to dispel myths.

This project led to a full programme that enables and supports the Indo-Asian community to tackle the challenge of diabetes in their population. The tactics used were so successful, it led to a re-orientation of the health services and the current community-based, culturally sensitive approach to managing chronic disease among the multicultural communities in Calgary today.

## Strengthen community action

Almost all public health nutrition community-based activities aim to strengthen community action, the third priority action area outlined in the Ottawa Charter. During the first few years of this new millennium in Calgary, Alberta, there was a particular focus on community-based childhood obesity prevention programmes for the early years from birth to school age^(^^33^^)^. Public health nutrition activities included supporting the implementation of good nutrition policies by day-care operators, facilitating food and healthy eating classes for ‘Mom and Toddler’ groups, providing a tele-advice line on nutrition and active living and hosting special media events such as infant feeding and healthy eating for pre-school children on breakfast television shows. Evaluation of how well these activities enabled communities to take action to improve nutrition was limited to collecting feedback on various events from those who attended. It was recognised that a more in-depth appraisal was required to assess overall impact on childhood obesity trends. The work described under this priority action concerns the development of a surveillance system of pre-school childhood weight status, which provided reliable data for assessing how effectively community action was being strengthened by the range of obesity prevention initiatives targeting the early years.

In 2000, the Centers for Disease Control and Prevention growth charts used in Canada were revised to include new sex-specific BMI-for-age charts which facilitated assessment of childhood overweight (these charts have since been updated to include WHO growth standard data^(^^34^^)^). Although public health nurses in the Calgary Health Region routinely measured children at vaccination visits and advised families on healthy growth, there was no formal assessment of weight status in terms of identifying children to be of healthy weight, or as under-, or overweight. Initially the idea of formally introducing growth assessment for obesity prevention at the pre-school vaccination visit was met with considerable resistance from public health nursing staff. This was mainly due to a lack of confidence among public health nurses in their ability to accurately assess growth and concerns about how to sensitively communicate on children's weight problems with families. In addition, many nurses cited being conscious they had weight problems themselves.

All objections raised by the public health nurses were well-founded and needed to be addressed. Through wide-ranging consultation the key elements required for successful implementation of growth assessment were identified. These elements included a protocol, training, equipment maintained for accuracy, counselling materials for parents, referral links with family physicians etc. (details outlined in^(^^35^^)^). All elements were put in place by a multidisciplinary team. A pilot was conducted in four of the eighteen health centres in the Region and this included evaluation of parent and public health nurse feedback on the acceptability and feasibility of the growth assessment protocol. This evaluation found a significant improvement in public health nursing confidence in providing growth assessment for children and in counselling parents. Parents rated the growth assessment system highly regardless of the weight status assigned to their children^(^^35^^)^.

Within a year this system was successfully implemented in all the eighteen health centres in the Region and the overall weight status of children assessed from February to December 2003 are outlined in Table 4. Approximately one quarter of children were found to be either at-risk of being overweight or were overweight; a slightly lower rate than in other parts of Canada. As 80 % of pre-school children attended for vaccination this system captures the weight status of most children in the Region. The model developed maximised the use of the existing resources and identified variation in weight status by location (each health centre area). This provided valuable information for public health planning and assessment of where resources were most needed to strengthen community action^(^^35^^)^.

**Table 4.** Weight status of children (*n* 7048, mean age 4·9 (sd 0·6) years) attending the Preschool Vaccination and Assessment Clinic at the Calgary Health Region, Community Health Centres (February 2003 – December 2003)^(^^36^^)^. (Re-produced with permission from The *Canadian Journal of Public Health* 2005; 96(6).)

Weight status (BMI-for-age percentile)*†	Girls % (*n*)	Boys % (*n*)	Total % (*n*)
Underweight (<5th)	2 (82)	3 (94)‡	3 (176)
Healthy weight (≥5th, <85th)	75 (2588)	72 (2583)	73 (5171)
At risk overweight (≥85th, <95th)	15 (509)	16 (562)	15 (1071)
Overweight (≥95th)	8 (260)	10 (370)	9 (630)
Total	49 (3439)	51 (3609)	100 (7048)

* BMI, kg/mg^2^.

† Centers for Disease Control and Prevention *et al.* (2000).

‡ Pearson's *χ*^2^ test = 20·26, *P* = 0·002 for sex differences in weight status.

## Create supportive environments

The fourth priority area for action, create supportive environments, outlined in the Ottawa Charter is all about making healthy options the easy options. This is what Mary wishes for when she imagines ‘a world where things are switched around, so that healthy eating and active living are easy and that, instead, people have to really struggle to have unhealthy lifestyle habits’ (see earlier). Calorie menu labelling is an example of public health nutrition action to create supportive environments to reduce obesity rates.

Research indicates that only about 15 % of consumers actively use calorie menu information at any given time; however, this does enable them to make lower calorie food choices^(^^36^^)^. Nonetheless, the 85 % of consumers who are not actively using the calorie information also benefit because calorie labelling creates a market for healthier food options. For example, the implementation of calorie menu labelling in America resulted in a fried chicken fast food chain introducing grilled options for the first time; and prompted a bakery chain to introduce smaller portions^(^^36^^,^^37^^)^. This indicates how calorie menu labelling in restaurant chains may help combat obesity even where only modest changes occur in consumer behaviour^(^^38^^)^.

**Fig. 2.**
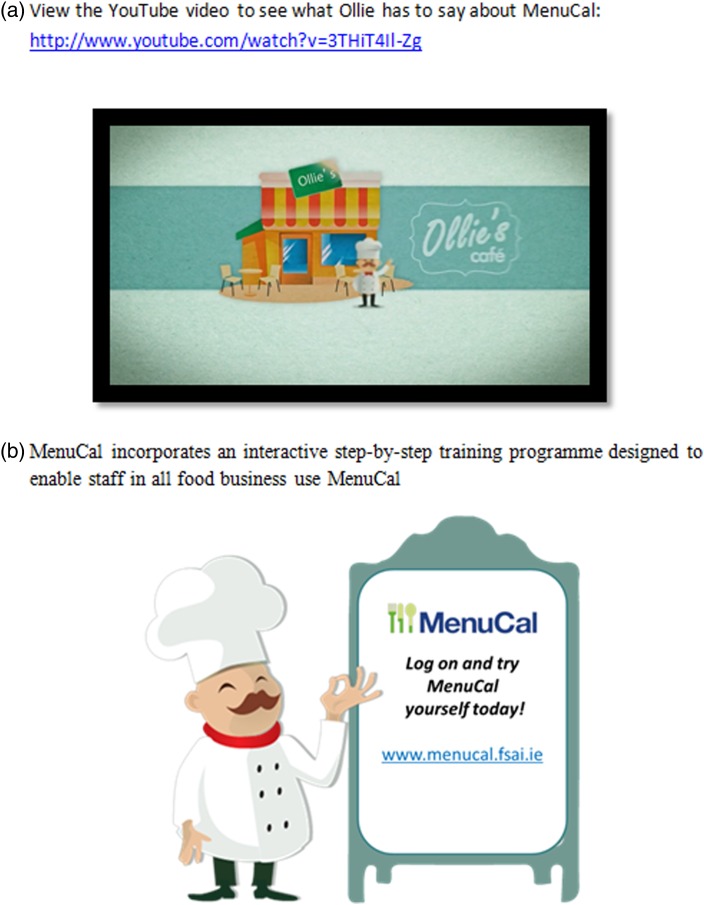
(a) and (b): Putting calories-on-menus in Ireland: Ollie the Chief tells you all about MenuCal - a calorie calculator designed to enable food businesses to calculate and display the calories in the food they serve.

The food environment in countries that have implemented calorie menu labelling tends to be dominated by big chains of food outlets. This situation enables the calculation and display of calories on the menus in one outlet to be replicated across the chain of twenty, or more, outlets, so costs per outlet are significantly diluted. The food environment in Ireland differs in that the majority of food business outlets are small, single enterprises that specialise in providing their own unique dishes. The challenges involved in putting calories on menus in this type of food environment are obviously much greater. However, the owner-operated, family-managed food service business sector in Ireland is run with a passion not seen in big chains. The creation of unique, award winning dishes and commitment to giving customers what they want are hallmarks of the Irish food service industry. Although initially resistant to the idea of voluntary calorie menu labelling^(^^39^^)^, the findings from a national public consultation that over 95 % of consumers in Ireland wanted to see calorie information in most food service outlets^(^^40^^)^ generated interest among food businesses^(^^41^^)^. The main reasons given by food businesses for not putting calorie information on their menus was the lack of skills they had to implement this and the high costs of hiring in necessary nutrition expertise both initially and in the long-term when menus change^(^^41^^)^. Evaluation of the quality of calorie menu labelling in Dublin found that the accuracy of calorie information was good but some improvements to the way the information was displayed would enhance the ease of use by consumers^(^^42^^)^. The development of a free on-line calorie calculator specifically for food service business personnel who have no nutrition background^(^^43^^)^ addresses these issues. MenuCal the calculator has been tested to ensure accuracy^(^^44^^)^ and has advanced functions to calculate fat absorbed during frying^(^^45^^)^. As shown in Fig. 2, MenuCal also has in-built interactive training to guide food business personnel through all the steps from getting their kitchens in order, through inputting recipes and displaying calorie information in the best way for ease of use by consumers^(^^43^^)^.

Consider the effects of posting calorie information on the food described earlier that Jayne and Mary enjoy on their trip to the cinema. The bucket of popcorn they share contains almost 300 g (296 g) popcorn and provides at least 4644 kJ (1110 kcal), while each of their jumbo soft drinks contain 1·4 litres of cola and provide 2460 kJ (588 kcal). Mary and Jayne's usual movie trip snack habits provide each of them with 4782 kJ (1143 kcal) amounting to about two-thirds of their entire calorie needs for the day. Displaying calorie information on such foods alerts motivated parents into avoiding these super-sized snacks for their children. Better still is the additional effect whereby calorie menu labelling creates a market for healthier food options that everyone, motivated or not, can benefit from. This creates a supportive environment for healthy eating that makes it easy, and probably even some fun too!

## Build healthy public policy

Build healthy public policy represents the most powerful and far-reaching of all the priority areas for public health action outlined in the Ottawa Charter because this tends to affect everyone. Mandatory fortification programmes such as folic acid food fortification to prevent birth defects (specifically neural tube defects (NTD)) that occur in early pregnancy, are excellent examples of building healthy public health nutrition policy.

The complexity of food fortification for specific health outcomes is demonstrated by Ireland's recent experience of folic acid food fortification^(^^46^^)^. Ireland has a history of high rates of pregnancies affected by NTD, 70 % of which are prevented by consumption of folic acid, the synthetic form of the B vitamin folate, prior to conception (see^(^^46^^)^ for review). In 2006, a national review of the evidence, in particular the effectiveness of folic acid food fortification in North America in reducing such birth defects, led to a policy decision to introduce mandatory folic acid food fortification in Ireland^(^^47^^)^.

However, the 2006 National Review raised questions about how much folic acid may already be in the Irish food supply. This was due to a noticeable increase in voluntary food fortification that started in 2003 when discussions at EU level began on the regulation of nutrition and health claims^(^^47^^)^. In addition, the national review highlighted a need to establish the baseline folate status of all population subgroups before implementing the mandatory fortification programme. It was recognised that this was the only way the true effects of implementing mandatory fortification could be assessed. This pre-mandatory fortification baseline assessment involved measuring blood folate status of women of child-bearing age, the target group, as well as other subgroups who would be exposed, particularly children and the elderly. It was recognised that this was important for evaluation of any potential overexposure. Finally, a comprehensive assessment of the prevalence of pregnancies affected by NTD was also conducted as rates had been declining since the 1980s, but the available data to assess this were incomplete^(^^47^^)^.

This baseline pre-mandatory fortification research found that 25 % of the children and 34 % of the elderly who provided blood samples already had very high blood folate levels and the incidence of deficient folate status among all groups had fallen from 5 % to <2 %^(^^46^^,^^47^^)^. In addition, the rate of pregnancies affected by NTD had also fallen to approximately one case per 1000 pregnancies, a level that was considered would not be improved by additional folic acid in the food supply^(^^46^^,^^47^^)^. The reasons for the improvement in folate status and the declining incidence of NTD, was related to increased amounts of folic acid in the food supply due to an escalation in voluntary folic acid food fortification. This was related to changes in food law at the EU level where voluntary food fortification was permitted^(^^48^^)^ and regulation of nutrition and health claims on foods meant scientific rationale (such as folate enrichment) was required for foods to bear claims^(^^49^^)^. Taking account of all of baseline research, including declining the numbers of pregnancies affected by NTD and high folate status evident among subgroups of the population that would not directly benefit from the mandatory fortification programme, led to a decision to postpone mandatory folic acid food fortification and to continue to monitor the situation^(^^46^^,^^47^^)^.

Since 2006 regular monitoring of voluntary food fortification (amounts added to foods and the range of food fortified), blood folate levels of subgroups of the population and the incidence of pregnancies affected by NTD in Ireland has been on-going. Through monitoring and enforcement actions the level of ‘overage’ (excessive folic acid added to foods relative to amounts indicated in food labelling) has been reduced in food and food supplements (M Flynn, personal communication). However, the range of foods voluntarily fortified with folic acid by food manufacturers continues to change and the amounts of folic acid added to fluctuate. This is the major disadvantage of voluntary fortification for public health needs because the types of foods fortified and the amounts of folic acid added are governed by business and marketing concerns rather than public health rationale. Furthermore, voluntarily fortified foods tend to be more expensive and this raises concerns that voluntary folic acid food fortification may not reach the more disadvantaged women, who are more vulnerable to folate deficiency and having a pregnancy affected by NTD. A recent report indicates the levels of pregnancies affected by NTD in Ireland has not reduced and is higher compared with countries that have mandatory folic acid food fortification in place^(^^50^^)^.

In summary, the Irish experience of folic acid food fortification indicates that for public health needs, mandatory fortification has many advantages over a voluntary approach. Nonetheless, voluntary folic acid food fortification has been in place for several decades and has certainly played a role in reducing pregnancies affected by NTD in Ireland. Finally, the work completed in this area of public health nutrition highlights the critical role of monitoring and evaluation to ensure expected benefits are realised, that there is minimal risk to those who do not benefit but are exposed and to assess when further action is warranted.

## Conclusions and evaluation

The present paper describes a range of nutrition initiatives as examples on how to take action in each of the five priority areas outlined in the Ottawa Charter. Although the present paper outlines action under just one priority area per initiative, in usual public health nutrition practice several priority areas feature simultaneously. Consider Mary, the 7-year-old child, and her family who have developed personal skills to eat healthily and be more active but are struggling to implement this. If a healthy eating/active living policy was built in her school and after school care settings, then this would create a supportive environment for a large part of Mary's day. Finally, this would also strengthen community capacity for healthy living in these settings. In conclusion, the inherent strength of the Ottawa Charter is how it drives a multi-strategic approach that empowers people to be healthier.

Evaluation and evidence-based decision-making is central to best practice in public health nutrition. In clinical settings the randomised control trial controls the situation to assess the true effects of interventions and this represents the gold standard approach to determine best practice. However, this approach needs adaptation to find out what works best in public health nutrition where all interventions play out in real life with all its confounding factors^(^^51^^,^^52^^)^. As shown in the present review, public health nutrition practice requires constant evaluation and research to gain an understanding of real-life complexities, so we can harness beneficial, and prevent detrimental confounding factors.
